# Electrochemical deposition of conductive and adhesive polypyrrole-dopamine films

**DOI:** 10.1038/srep30475

**Published:** 2016-07-27

**Authors:** Semin Kim, Lindy K. Jang, Hyun S. Park, Jae Young Lee

**Affiliations:** 1 Gwangju Institute of Science and Technology, School of Materials Science and Engineering, Gwangju, Gwangju 500-712, Republic of Korea; 2 Korea Institute of Science and Technology, Fuel Cell Research Center, Hwarangro 14-gil 5, Seoul 02792, Republic of Korea; 3 Gwangju Institute of Science and Technology, Department of Biomedical Science and Engineering, Gwangju, Gwangju 500-712, Republic of Korea

## Abstract

Electrode surfaces have been widely modified with electrically conductive polymers, including polypyrrole (PPY), to improve the performance of electrodes. To utilize conductive polymers for electrode modification, strong adhesion between the polymer films and electrode substrates should be ensured with high electrical/electrochemical activities. In this study, PPY films were electrochemically polymerized on electrodes (e.g., indium tin oxide (ITO)) with dopamine as a bio-inspired adhesive molecule. Efficient and fast PPY electrodeposition with dopamine (PDA/PPY) was found; the resultant PDA/PPY films exhibited greatly increased adhesion strengths of up to 3.7 ± 0.8 MPa and the modified electrodes had electrochemical impedances two to three orders of magnitude lower than that of an unmodified electrode. This electrochemical deposition of adhesive and conductive PDA/PPY offers a facile and versatile electrode modification for various applications, such as biosensors and batteries.

Electrically conductive polymers, such as polypyrrole (PPY), have been extensively studied as active electrode materials and supporting additives for a number of applications, including electronics, batteries, electrodes, and biomaterials^1^,^2^,^3^,^4^,^5^. PPY is a widely studied conductive polymer because of its high conductivity, chemical stability, and biocompatibility^6^,^7^. Thus, PPY has been widely used for electrode surface modification to improve electrode performance. In particular, electrochemical deposition offers a versatile and facile method for the synthesis of PPY films on electrodes because the film formation and its properties (e.g., thickness and surface topography) can be easily controlled by the electrochemical polymerization conditions (e.g., electrode potential, current density, and electrolyte solutions)^8^,^9^. However, the adhesion of PPY coatings on electrode surfaces is poor due to the lack of strong molecular interactions between PPY and the electrode, which greatly hinders the practical applications of PPY-based electrode modification^10^. Many researchers have attempted to improve the adhesion and electrical properties of PPY films with various approaches, such as pre-treating electrodes^11^,^12^,^13^, selecting proper polymerizing solvents^14^,^15^, introducing interfacial adhesive layers^16^, and modifying the pyrrole derivatives^17^,^18^,^19^,^20^. However, the majority of attempts caused several critical issues, such as insufficient improvement in the mechanical adhesion of the PPY film^21^, preparation difficulties^22^, or severe impairment of the electrical performance of the electrodes^23^. Consequently, the development of an efficient PPY coating technique for mechanically adhesive and electrochemically superior conductive electrodes is strongly desired.

Dopamine (DA) is a bio-inspired adhesive molecule that mimics the critical bio-adhesive moieties of marine mussels^24^. This molecule and its functionalized substances can be used to modify material surface properties and to improve coating adhesion^25^. DA can be readily deposited on substrates via self-polymerization to polydopamine (PDA), which strongly interacts with a variety of materials (e.g., metals, metal oxides, polymers, ceramics) in a substrate-independent manner^24^,^26^,^27^. PDA formation can be typically induced by shifting the solution pH to alkaline or by enzymatic oxidation^24^. Several other studies demonstrated the electrochemical deposition of PDA on conductive electrodes^16^,^28^. Ji *et al*. electrochemically coated PDA on gold stents and demonstrated easy control of the coating degree using an electropolymerization method in contrast to the conventional chemical PDA self-polymerization^29^. However, PDA is not electrically conductive and forms an insulating layer on an electrode. Therefore, the electrochemical deposition of either PDA or PPY alone could not meet two important properties associated with electrode performances: adhesion and electrical conductance^30^,^31^. Recently, Zhang *et al*. chemically polymerized PPY nanoparticles with DA using chemical oxidant ammonium persulfate. They found that the films, prepared with the DA-incorporated PPy nanoparticles, showed higher electrical conductivity and the greatly improved adhesion to glass substrates compared to the films prepared with PPY nanoparticles. However, the use of chemically synthesized PPY-DA nanoparticle is not suitable to modify electrode surface due to the inherent difficulties in finely controlling layer thickness and selectively coating them on electrode surfaces^32^,^33^. In this study, with the aim of improving electrode performance, we electrochemically polymerized PPY with DA to form conductive coatings, PDA/PPY, on ITO electrodes by utilizing the electrically conductive polymer PPY and mussel adhesive-inspired DA chemistry (Fig. 1). Synthesis conditions of PDA/PPY and characteristics of the electrochemically produced coatings and electrodes were investigated.

## Results

### Electrochemical deposition of PDA/PPY films

To study the oxidation of the monomers, cyclic voltammetry (CV) tests of DA, pyrrole, and DA/pyrrole on ITO electrodes were performed in phosphate-buffered saline (PBS, pH 6) (Fig. 2a). PBS was employed as biologically relevant electrolyte buffer solutions, containing chlorine and phosphate anions. The solution pH was maintained to 6 to prevent spontaneous DA oxidation to PDA because DA oxidation is strongly affected by the solution pH and DA spontaneously polymerizes at slight alkaline pH. From the cyclic voltammogram of DA (26 mM), the multiple peaks were observed. The anodic peak at 0.27 V and the cathodic peak at 0.03 V were assigned to the oxidation of DA to dopaquinone (DQ) and the DQ reduction to DA, respectively^29^,^34^. The current at the working electrode (i.e., ITO) during CV remained low (<120 μA) and even decreased slightly with an increasing number of cycles, indicating inefficient polymerization and insulating layer formation. Pyrrole (150 mM) did not substantially polymerize during the first two cycles but eventually began to polymerize at values above 0.5 V. By contrast, the DA/pyrrole mixed solution (130 mM pyrrole and 26 mM DA, [DA]/[pyrrole] = 0.2) exhibited a broad anodic peak at 0.2 V and a large current from the first cycle, which also increased with an increasing number of cycles, indicating that the oxidative polymerization of pyrrole and DA (PDA/PPY) was facilitated. In addition, electrodeposition was performed by chronoamperometry at 0.5 V (vs. SCE) (Fig. 2b). Pyrrole (150 mM) did not substantially lead to polymerization under this condition. By contrast, the DA/pyrrole monomer solution (130 mM pyrrole and 26 mM DA) led to substantial oxidation with large current (>0.1 mA), indicating a catalytic role of DA.

The effects of DA on electrochemical pyrrole polymerization on ITO were further investigated by varying the DA concentrations (Table 1). As the DA concentrations (DA/pyrrole molar ratios) increased, the oxidation currents increased accordingly, indicating greater and faster polymerization of PDA/PPY (Fig. 2c). The masses of the coated polymers on the electrodes during chronoamperometry (at 0.5 V) were also measured using an electrochemical quartz crystal microbalance (EQCM). The results revealed continuous growth of the films over time in all samples (Fig. 2d). Interestingly, mass deposition increased as the DA/pyrrole ratio increased to 0.2 in the monomer solution. The mass deposition rate gradually decreased at higher DA concentrations (e.g., 52 mM or 130 mM DA) than this ratio. At the higher DA concentrations, PDA/PPY preferentially polymerized in solution in the vicinity of the electrode surfaces rather than on the top of the electrode, likely due to the rapid electron transfer and pyrrole oxidation, and thus resulted in thin coatings on electrodes (Supporting Information Figure S1).

### Characterization of PDA/PPY films

Raman spectroscopic analysis was performed to examine dopamine incorporation into the conductive polymer films during electrodeposition (Fig. 3). The results indicated the presence of DA in PDA/PPY films with characteristic DA-associated peaks. The peaks at 1040 cm^−1^ and 1567 cm^−1^ are assigned to C-O stretching of DA and in-plane aromatic ring bending of DA according to previous literatures^35^,^36^,^37^. These results confirmed the incorporation of DA during electropolymerization with pyrrole. This incorporated DA molecules are expected to play important roles in adhesion improvements and electrochemical characteristics of the produced PDA/PPY films.

**Figure 1:**
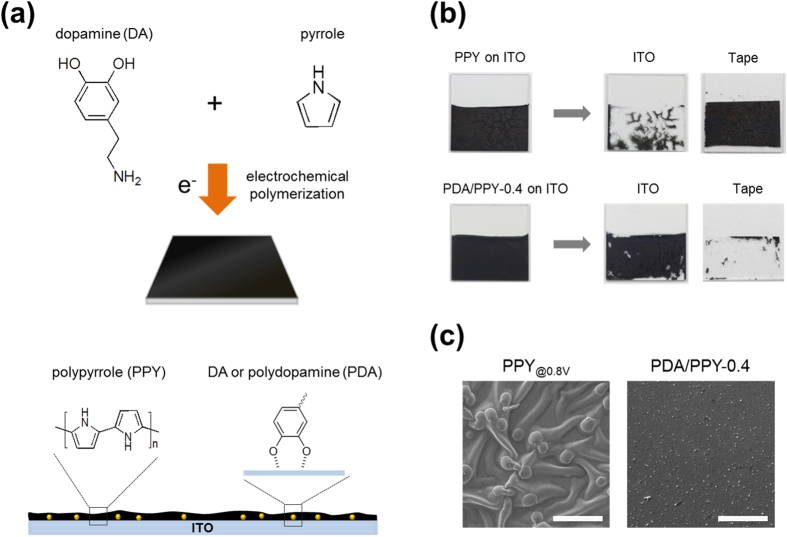
Electrochemical synthesis of conductive polymer films (PDA/PPY) with pyrrole and dopamine on electrodes. (**a**) Schematic illustration of the electrodeposition of PDA/PPY, in which DA moieties improve adhesion of the coating and the electrode performance. (**b**) Photographs of the PPY- or PDA/PPY-coated ITO electrodes (2.5 × 2.5 cm) before and after the Scotch tape detachment test. (**c**) Scanning electron micrographs of PPY and PDA/PPY. PPY and PDA/PPY samples were electrochemically synthesized on ITO at 0.8 V and 0.5 V (vs. SCE), respectively, for 300 s. Scale bars represent 50 μm.

**Figure 2:**
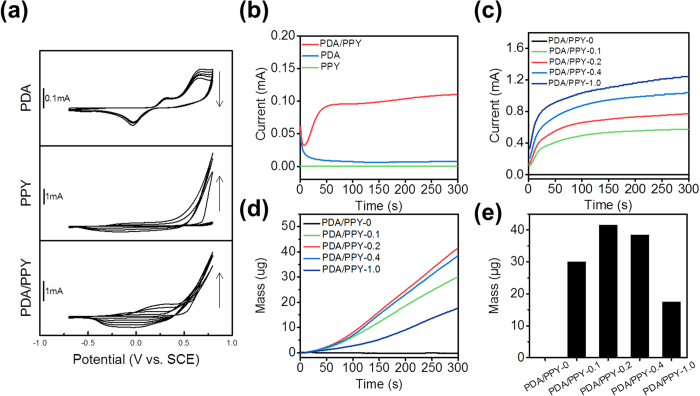
Electrochemical synthesis of PDA/PPY films on electrodes. (**a**) CVs of the monomer solutions containing DA (26 mM), pyrrole (150 mM), and DA/pyrrole (26 mM DA and 130 mM pyrrole) in PBS (pH 6). (**b**) Chronoamperometry (0.5 V vs. SCE) in the pyrrole and/or DA solutions on ITO electrodes with the same condition of (a). (**c**) Chronoamperometry (0.5 V) in various monomer solutions (see the Table 1 for the compositions). (**d**) Electrochemical QCM profiles during chronoamperometry (0.5 V) of the various monomer solutions (see Table 1 for the compositions). (**e**) Mass deposited on electrodes during electrodeposition.

The adhesion strengths of the electrochemically synthesized PDA/PPY films on ITO electrodes were measured (Fig. 4a,b). The control PPY films (PPY_@0.8V_) were synthesized at 0.8 V (vs. SCE) and used because the PPY homopolymer (PDA/PPY-0) could not be obtained at 0.5 V. The control PPY films were not adhesive and easily delaminated from the electrodes even in a mild washing step; thus, its adhesion strength could not be measured. The PDA/PPY films could not be removed by the Scotch tape, whereas the PPY_@0.8V_ film was entirely detached (Fig. 1b). Adhesion strengths of the PDA/PPY films were greatly promoted to >2.5 MPa. Figure 4 shows that the adhesion strengths gradually increased as the DA concentration in the synthetic solution increased. The PDA/PPY-1.0 films exhibited the highest strength of 3.7 MPa. In addition, PPY-coated electrodes showed distinct micro-wrinkle structures resulting from local delamination, whereas the PDA/PPY film surfaces were smooth and stably attached without such buckling (Supporting Information Figures S2 and S3). The improved adhesion of the conductive films without buckling of the PDA/PPY films is believed to result from the adhesive DA moieties presented in the films that can strongly interact with electrode surfaces. Hence, the PDA/PPY films strongly adhered onto the electrode were expected to allow for stable and improved electrode performance.

The electrical and electrochemical properties of the PDA/PPY-modified electrodes were characterized. The electrochemical impedance spectra of the PDA/PPY films were obtained in PBS (pH 7.4) as the low impedance and high conductivities are important requirements for various applications, such as bioelectrodes. PDA/PPY-coated ITO electrodes exhibited low impedance, especially at low frequencies (1–1,000 Hz) (Fig. 5a, Supporting Information Figures S4). Impedances of the PDA/PPY electrodes were two to three orders of magnitudes lower compared with that of the bare ITO. Additionally, these values are considerably lower than that of the PPY-coated ITO. For example, the impedances of PDA/PPY electrodes were 73–120 Ohm at 1 Hz and 70–76 Ohm at 10 Hz, whereas unmodified ITO electrodes and PPY electrodes had impedances of 62,000 Ohm at 1 Hz and 68,000 Ohm at 10 Hz and 73–280 Ohm at 1 Hz and 271 Ohm at 10 Hz, respectively. The PDA/PPY-0.2 electrode showed the lowest impedance among the various PDA/PPY-coated electrodes. Moreover, the conductivities of the PDA/PPY films were higher compared with that of the PPY_@0.8V_ film (Fig. 5b). As the DA concentrations increased during electrodeposition, the film conductivity increased to 0.28 ± 0.05 S cm^−1^ (PDA/PPY-0.2) from 0.12 ± 0.02 S cm^−1^ (PPY_@0.8V_) and then began to decrease to 0.03 ± 0.02 S cm^−1^ (PDA/PPY-1.0). Among the various modified electrodes, the PDA/PPY-0.2 showed the best electrical/electrochemical activities. Thus, the electrochemical characteristics and the stabilities of PDA/PPY-0.2 electrode and the control PPY_@0.8V_ electrode were studied with CV using a redox reagent, K_3_Fe(CN)_6_ (Fig. 5c). The results indicated that the PDA/PPY-0.2 film was conductive and sufficiently electrochemically active to support a reversible redox reaction. For example, the PDA/PPY-0.2 electrodes had 3.9-fold higher charge capacity compared to that of the control PPY_@0.8V_ electrodes at the second cycle, likely due to a large electrode surface area and efficient electron transfer at the PDA/PPY-0.2 film surfaces. For the control PPY_@0.8V_ electrodes, the conductive films were readily detached and maintained a low capacitance during the CVs. In addition, the PDA/PPY-0.2 electrodes exhibited relatively good stability during the CV tests. After 100 cycles, the electrodes’ capacitance was 83% of the initial value.

## Discussion

With the aim of improving electrode properties (e.g., conductance and adhesion), PDA/PPY films were electrochemically polymerized on electrodes using the electrically conductive polymer PPY and mussel adhesive-inspired DA. In our study, the addition of DA in the electrochemical PPY polymerization process greatly increased the reaction rates. Importantly, the adhesion and electrochemical properties of the modified electrodes were significantly improved. Therefore, DA was assumed to play multiple roles in electropolymerization processes and the characteristics of the resultant polymer films, which include (i) a catalytic effect on PPY polymerization, (ii) homogeneous initial deposition, (iii) improved adhesion and electrical conductivity of the PDA/PPY films. First, DA has a catalytic effect on the electropolymerization of PPY films. In the presence of DA, conductive films could be grown at lower potential (>0.4 V) than the potentials (~0.8 V) in the absence of DA. DA is oxidized to DQ. Then, DQ reacts with the pyrrole monomer to produce a radical pyrrole cation, of which series reactions can accelerate the pyrrole radical formation and polymerization. The generation of pyrrole radicals from pyrrole monomers is known to be difficult during PPY polymerization^38^. Catechol (CA), which has a quinone structure similar to that of DA, could also facilitate the electropolymerization of pyrrole (Supporting Information Figure S5). The results imply that compounds bearing a CA moiety can play catalytic role in pyrrole oxidation. In addition, homogeneous initial deposition was observed on the electrode surfaces, which might be attributed to the good interactions between DA and the electrode surface allowing for the homogeneous deposition of conductive polymers. In the presence of DA, initial deposition was found over the entire electrode surfaces and formed a smooth and homogeneous layer. On the other hand, in the absence of DA, initial PPY deposition occurred at a few sites as nuclei and then grew radially and formed a rough and heterogeneous deposition (Supporting Information Figure S6). PPY initial deposition is generally recognized as a rate-determining step of PPY film polymerization^38^. A surface morphology analysis revealed that the microstructural morphologies of the fabricated films were affected by the presence of DA (Supporting Information Figures S2 and S3).

**Figure 3:**
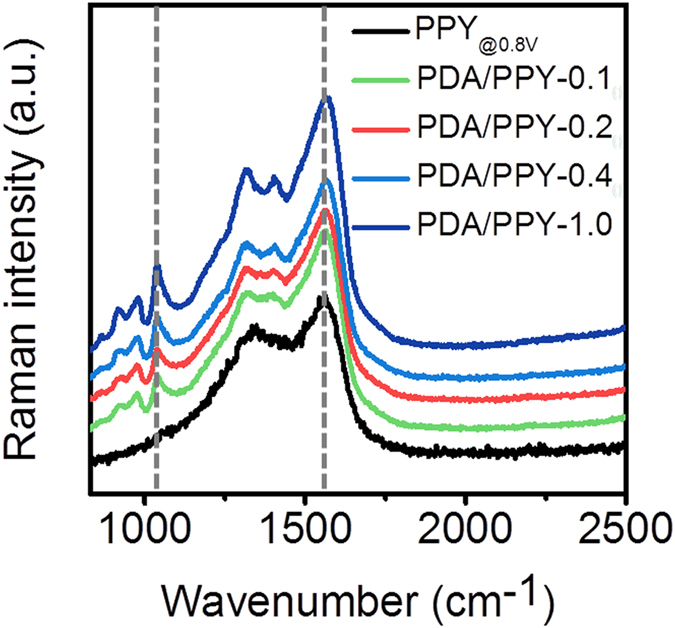
Raman spectra of PPY and PDA/PPY films electrochemically deposited at different molar ratios.

**Figure 4:**
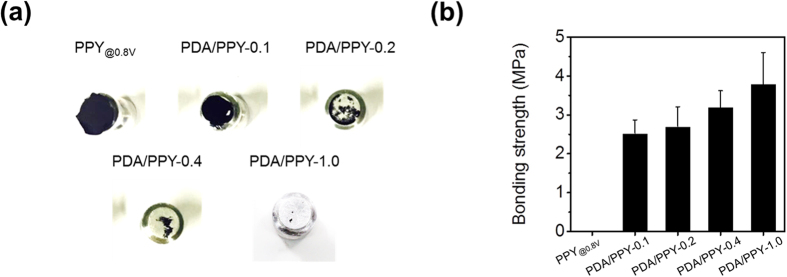
Measurement of the pull-off adhesion strength of the electrochemically synthesized PDA/PPY films. (**a**) Photographs of the dollies after the pull-off tests. (**b**) Bonding strengths of the PPY control and various PDA/PPY films on ITO electrodes.

**Figure 5:**
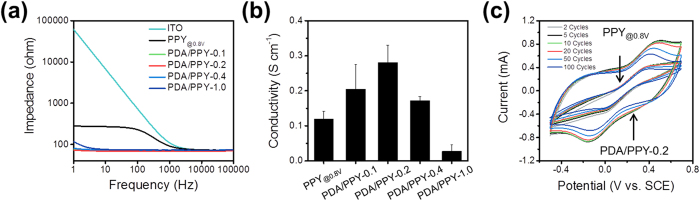
Electrochemical and electrical properties of PDA/PPY-coated ITO electrodes. (**a**) Electrochemical impedance spectra of PDA/PPY-coated ITO electrodes. (**b**) Conductivities of the PDA/PPY films (n = 3). (**c**) CVs of PDA/PPY-0.2 and PPY_@0.8V_ coated electrodes in a 10 mM K_3_Fe(CN)_6_ solutions for 100 cycles.

DA was incorporated into PPY chains to form copolymers during electropolymerization as shown in Raman spectroscopy (Fig. 3). However, it is still unclear whether DA was incorporated into the PPY chains as copolymers or PDA clusters were embedded in PPY. Systemic studies will be needed to the molecular structure of the fabricated PDA/PPY film as a crucial assignment for future studies to clearly understand the mechanisms of film formation and characteristics. Still, the incorporated DA could have positive effects on adhesiveness and electrochemical properties of the films. DA can form firm complexes with metal oxide (e.g., ITO) and lead to strong adhesion^39^. It should be noted that the films electrochemically polymerized using CA (a DA derivative) were brittle and lacked mechanical adhesiveness, indicating that the DA moieties incorporated in the PDA/PPY resulted in the strong film adhesion. In addition to adhesion, DA incorporation during the electropolymerization of PPY influenced the electrical properties of the PDA/PPY. Interestingly, the conductivities of the PDA/PPY films were affected by the DA concentration. PDA/PPY-0.1 and PDA/PPY-0.2 exhibited higher conductivities than the PPY films. However, PDA/PPY films synthesized at higher DA concentration (>26 mM) had lower conductivities. The incorporation of DA into PPY chains in moderate degrees might prevent random α-β pyrrole coupling, which impairs the conjugated π-orbital structures of the PPY backbone and causes a decrease in conductivity^32^, and therefore encourages the conductive α-α pyrrole coupling. By contrast, higher DA incorporation in PDA/PPY forms substantially non-conductive PDA and/or leads to shorter PPY chains, which can lower the conductivity of PDA/PPY^40^,^41^. The electrical impedances of all of the PDA/PPY-modified samples were considerably lower than those of the unmodified ITO electrodes and PPY-modified electrodes, particularly at low frequencies. These low impedances imply sufficient electron/charge conductivities of PDA/PPY films with enlarged electrode surface areas. This remarkable impedance improvement can be further explained by stronger film adhesion and conductive interfaces between the PDA/PPY films and ITO. The firm adhesion of PDA/PPY deposits on electrodes plays a decisive role in minimizing the local delamination of conductive coating layers and thus secures the utilities of conductive polymers as electrically active coating layer materials. Overall, regarding the greatly lowered impedances of the PDA/PPY-modified electrodes, these factors would enhance the coating conductivity and adhesion.

## Conclusion

Pyrrole and DA are electrochemically polymerized together to obtain electrically conductive and mechanically stable coatings for conductive polymers. DA was found to play a catalytic role enabling rapid and efficient deposition even at low potentials. In addition, DA-incorporated films showed greatly enhanced adhesion forces onto ITO electrodes up to 3.7 MPa. The synthesized films exhibited significantly enhanced conductivities to 0.28 S/cm and impedances 2–3 orders of magnitudes lower than that of unmodified or pristine PPY-coated electrodes due to the improved electrical and mechanical characteristics of PDA/PPY. This PDA/PPY conductive polymer-coated electrode displaying good adhesion and conductance will be greatly useful for various applications, including in batteries, electronics, and bioelectrodes.

## Methods

### Electrochemical Polymerization

Pyrrole (Sigma) was purified before use by passing it through an aluminum oxide (Sigma) column. The polymerization solution was prepared by mixing 0.15 M aqueous pyrrole in PBS (pH 6, Life Technologies) and aqueous DA solutions (PBS, pH 6) of different dopamine hydrochloride concentrations (Sigma). Compositions are listed in Table 1. Electrochemical deposition was performed in a three-electrode set-up using a VersaSTAT3 electrochemical working station (Princeton Applied Research) with ITO electrode as a working electrode (0.6 mm in diameter), platinum wire as a counter electrode, and a saturated calomel electrode (SCE, CH Instrument, Inc.) as a reference electrode. CV was performed in the polymerization solution using an ITO working electrode from −0.7 to +0.8 V (vs. SCE) at a scan rate of 0.02 V/s. For chronoamperometric polymerization, a constant voltage of 0.8 V (vs. SCE) for conventional PPY films and 0.5 V (vs. SCE) for PDA/PPY films were applied for 300 s at room temperature. The modified electrodes were washed with a copious amount of deionized water and dried at room temperature. To ensure a constant electrode area for polymerization and analysis, insulating tape (3 M) was punched to have a 6 mm well, and the tape was carefully placed onto the electrode. Electrochemical quartz crystal microbalance (EQCM) was performed on the mass deposited on the electrode during the electropolymerization process using a QCM instrument (QCM922A, Seiko EG&G) and ITO electrodes (CH Instrument).

### Scanning electron microscopy (SEM)

The morphologies of the PDA/PPY films were analyzed using field emission scanning electron microscopy (FE-SEM, Hitachi S-4700, Japan). Images were acquired without a metal coating because the samples were all electrically conductive.

### Adhesion test

A pull-off adhesion test was performed with PosiTest Adhesion Tester (PosiTest AT-T). A dolly with a 10 mm diameter was glued vertically onto the surface of the PDA/PPY substrates using an epoxy adhesive (Locstar), and the adhesive was left for curing overnight. After removing the excess adhesive, dollies were pulled off from the substrate, and the force needed to detach films from the ITO glass was measured. Three samples were tested on each sample.

### Cyclic voltammetry

The electrochemical properties of the PDA/PPY substrates were investigated by CV using a VersaSTAT3 electrochemical working station in a 10 mM K_3_Fe(CN)_6_ solution (dissolved in 0.1 M KCl) at a 0.1 V/s scan rate.

### Conductivity measurement

The electrical conductivity of PDA/PPY was measured using a four-point probe method (Modysystems, Korea). As-prepared films were immersed in 1 M HCl (Sigma) for 10 min to detach films from the ITO glass and obtain intact freestanding films. The detached films were thoroughly washed in deionized water and carefully placed on clean glass slides. After completely drying the films, linear scan voltammetry was applied from −1 V to 1 V. The resistance was measured from the I–V curve, and the film thickness was measured using a stylus profiler (Dektak XT, Bruker). The conductivity was then calculated from the film thickness and resistance according to the literature^42^.

### Electrochemical impedance spectroscopy (EIS)

The electrochemical impedance spectra of the PPY or PDA/PPY-modified electrodes were measured and compared with unmodified ITO electrodes using a VersaSTAT3 electrochemical working station. A three-electrode setup was employed as described above. Experiments were performed in PBS (pH 7.4) with an alternative sinusoidal potential of 10 mV and a DC potential of 0 V (vs. SCE) in the range of 1–10^5^ Hz.

### Raman spectroscopy

Raman spectra of PDA/PPY films were obtained by an in-plus Raman spectrometer (a UniThink Inc., UniRaman, Korea) with a 514 nm laser. The exposure time was set to 2 s and 6 s for PDA/PPY films and PPY films, respectively, because of low signals from PPY films.

## Additional Information

**How to cite this article**: Kim, S. *et al*. Electrochemical deposition of conductive and adhesive polypyrrole-dopamine films. *Sci. Rep.* **6**, 30475; doi: 10.1038/srep30475 (2016).

## Supplementary Material

Supplementary Information

## Figures and Tables

**Table 1:** Names of the various PDA/PPY samples and composition of the monomer solutions for electrodeposition^a^.

Sample name	[DA]_0_ (mM)	[pyrrole]_0_ (mM)	[DA]_0_/[pyrrole]_0_
PDA/PPY-0.1	13	130	0.1
PDA/PPY-0.2	26	130	0.2
PDA/PPY-0.4	52	130	0.4
PDA/PPY-1.0	130	130	1.0
PDA/PPY-0	0	150	0
PPY_@0.8V_^b^	0	150	0

^a^Electrochemical deposition was performed by chronoamperometry at 0.5 V vs. SCE for 5 min.

^b^PPY_@0.8V_ was produced by chronoamperometry at 0.8 V vs. SCE for 5 min.
