# Supplementary material for: Electrochemical deposition of conductive and adhesive polypyrrole-dopamine films
Source: Sci Rep. 2016 Jul 27;6:30475. doi: 10.1038/srep30475 (PMC4962031; doi:10.1038/srep30475)
Supplement: Supplementary Information [file srep30475-s1.pdf]

Supporting Information

Electrochemical deposition of conductive and adhesive polypyrrole-dopamine films

Semin Kim[1,+], Yesul Jang[1,+], Hyun S. Park[2], and Jae Young Lee[1,3*]

[1]Gwangju Institute of Science and Technology, School of Materials Science and Engineering, Gwangju, Gwangju 500-712, Republic of Korea

[2] Korea Institute of Science and Technology, Fuel Cell Research Center, Hwarangro 14-gil 5, Seoul 02792, Republic of Korea

[3]Gwangju Institute of Science and Technology, Department of Biomedical Science and Engineering, Gwangju, Gwangju 500-712, Republic of Korea

* jaeyounglee@gist.ac.kr, Fax: +82-62-715-2324, Tel: +82-62-715-2358

[+] These authors contributed equally to this work

Contents:

Figure S1. Thickness of PDA/PPY samples using Surface profiler.

Figure S2. SEM images of PDA/PPY samples.

Figure S3. Three dimensional surface morphologies of PDA/PPY samples.

Figure S4. Electrochemical impedance spectra of PDA/PPY-coated ITO electrodes.

Figure S5. Chronoamperometric plot for electropolymerization in various CA(catechol) and pyrrole solutions on ITO at a 0.5 V (vs SCE) constant potential.

Figure S6. Photographs of the PPY or PDA/PPY coated ITO electrodes electrochemically polymerized for different times.

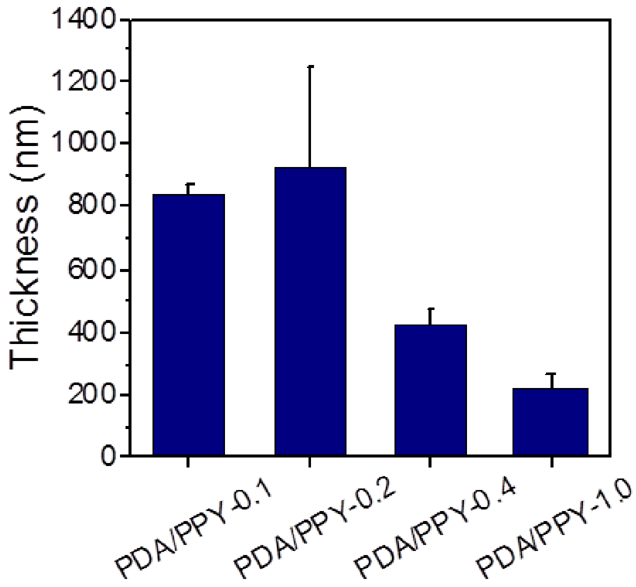

Figure S1. Thickness of PDA/PPY films synthesized on ITO at 0.5 V for 5 min. After the electrodeposition, the films were washed with double-deionized water and dried. Film thickness was measured using a stylus profiler (Dektak XT, Bruker). Three samples were used for the measurement (n=3). Error bars indicate the standard deviations.

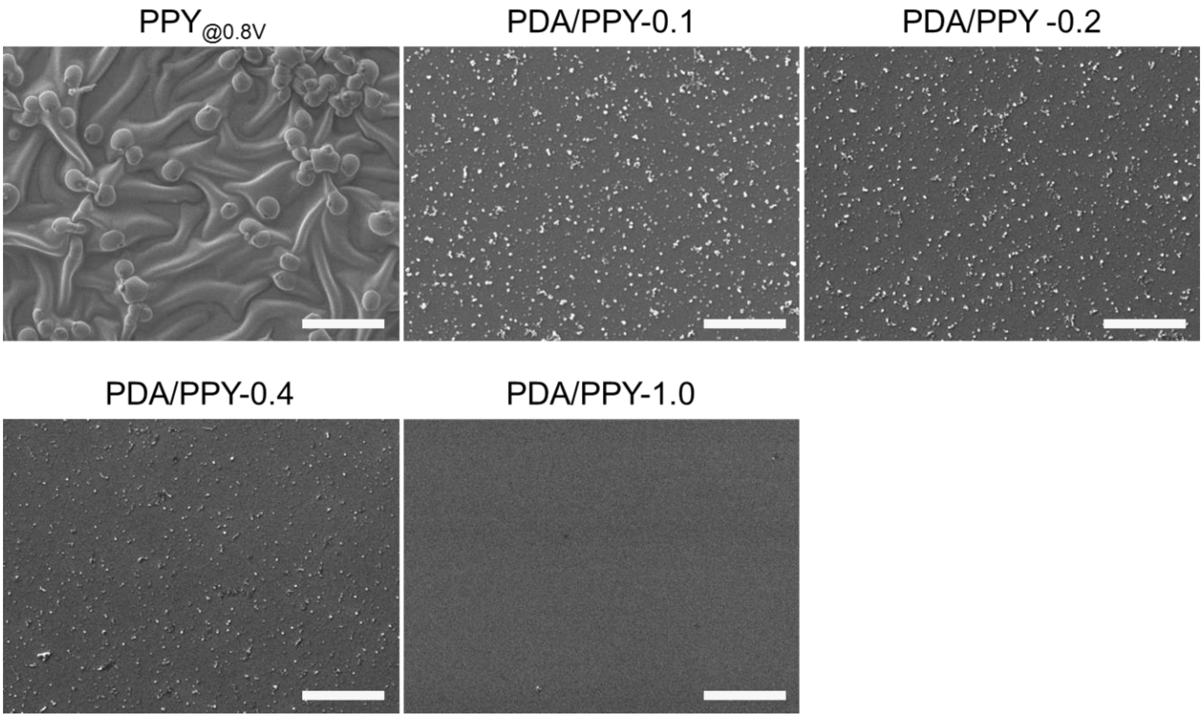

Figure S2. SEM images of PDA/PPY samples. SEM images were obtained with a field emission scanning electron microscope (FE-SEM, Hitachi S-4700, Japan). Samples for SEM analysis were dried in desiccator for 2 d. Samples were measured without metal coating. Scale bars represent 50 μm.

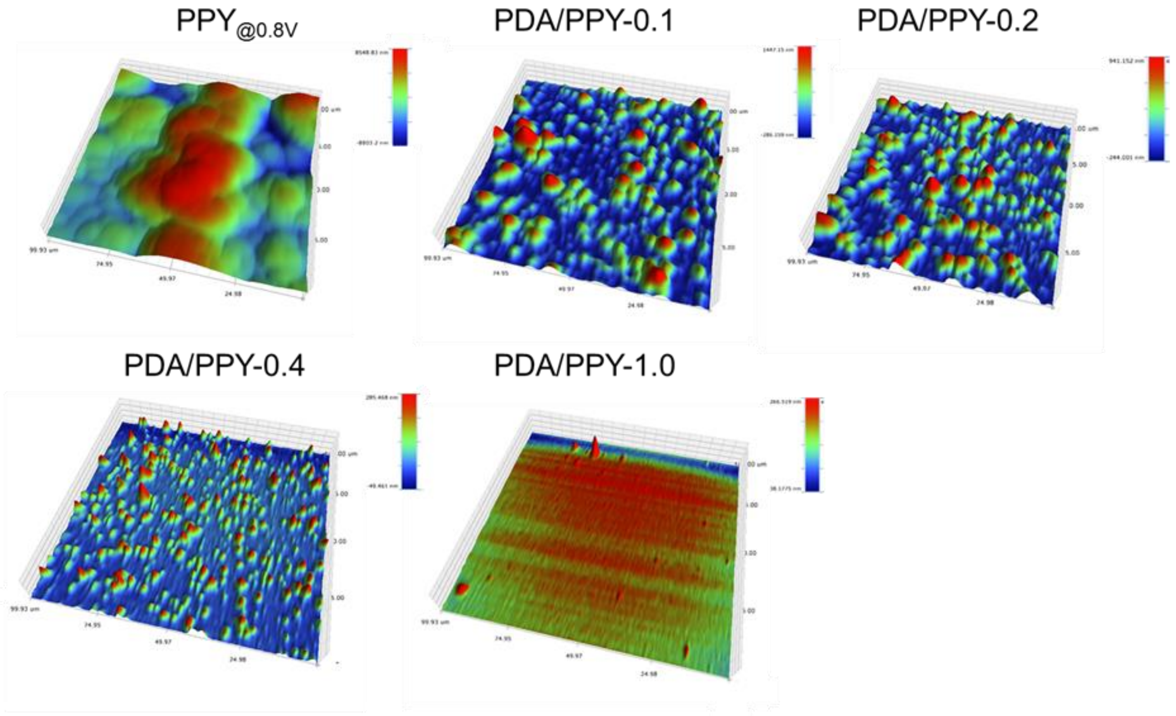

Figure S3. Three dimensional surface morphologies of PDA/PPY samples. Images and roughness were obtained by stylus profiler (Dektak XT, Bruker) Surface morphology of PDA/PPY samples were measured by stylus profiler (Dektak XT, Bruker) using 3D map scan mode with 12.5 μm stylus type. The films were completely dried at room temperature, and their surface areas of 100 X 100 μm were analyzed.

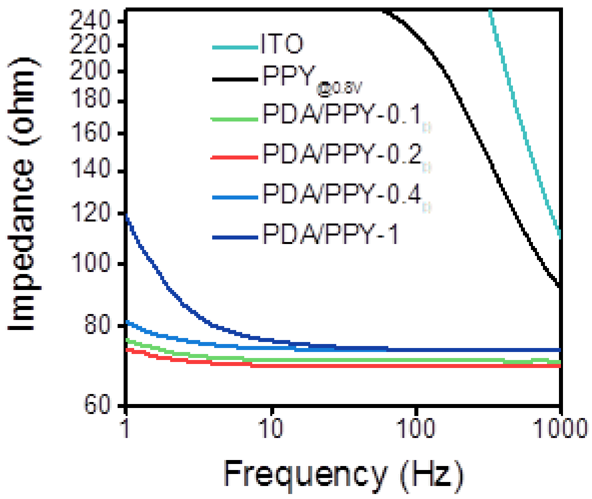

Figure S4. Magnified electrochemical impedance spectra of PDA/PPY-coated ITO electrodes. Experiments were performed in PBS (pH 7.4) at an alternative sinusoidal potential of 10 mV and DC potential of 0 V (vs. SCE) from 1 Hz to 100,000 Hz.

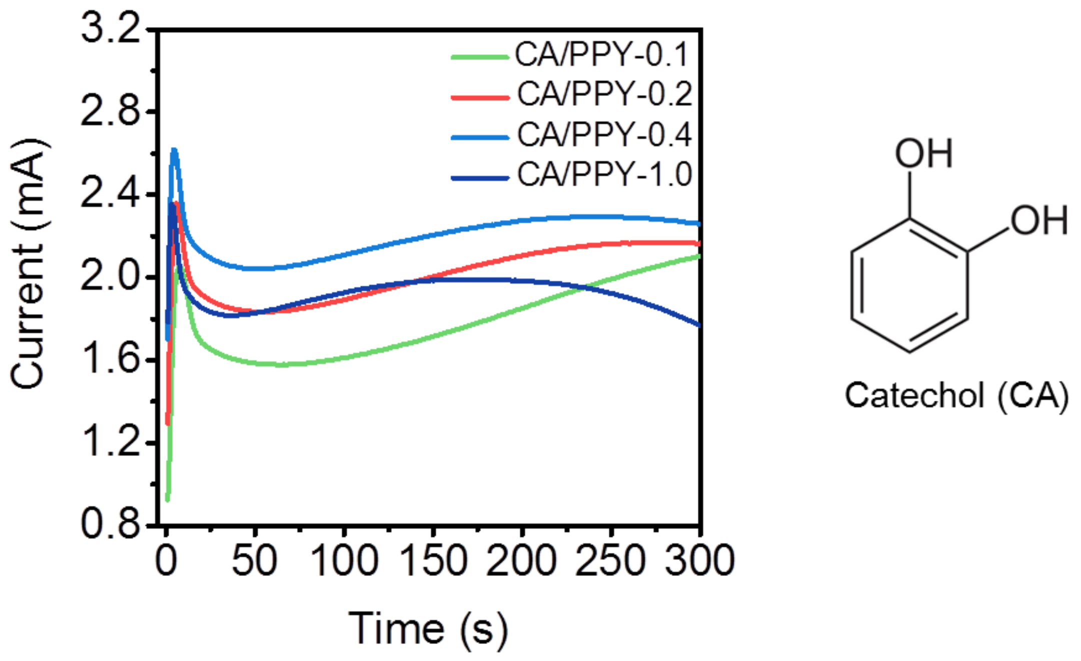

Figure S5. Chronoamperometric plot for electropolymerization in various CA(catechol) and pyrrole solutions on ITO at a 0.5 V (vs. SCE) constant potential. Concentrations of CA in pyrrole solution (130 mM in PBS, pH 6) were varied to have 13, 26, 52, and 130 mM, of which synthesized CA/PPY films were noted as CA/PPY-0.1, CA/PPY-0.2, CA/PPY-0.4, and CA/PPY-1.0, respectively.

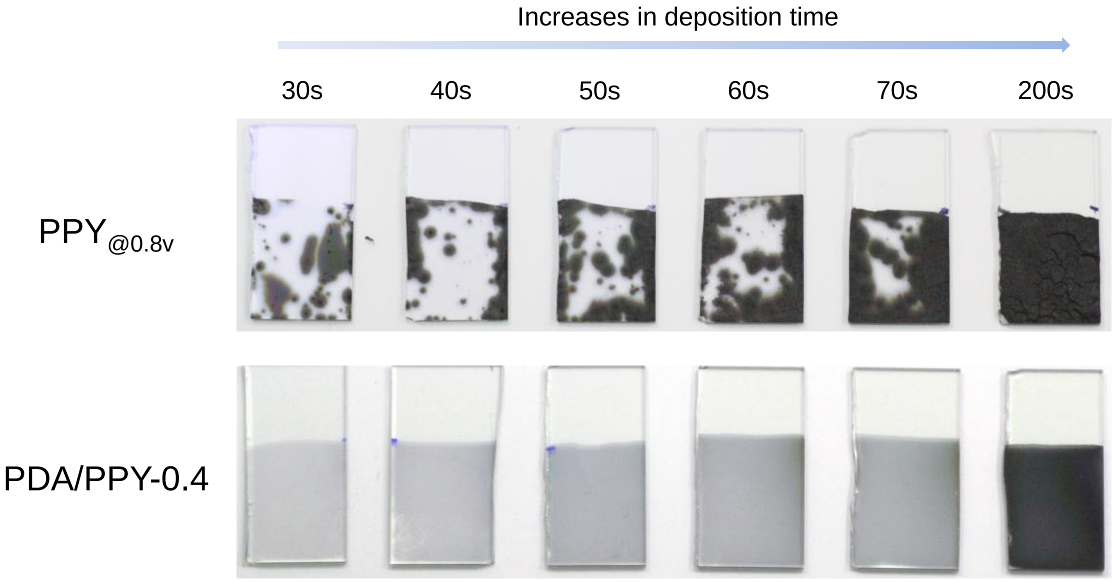

Figure S6. Photographs of the PPY or PDA/PPY coated ITO electrodes electrochemically polymerized for different times. Films were deposited in pyrrole solutions (130 mM in PBS, pH 6) or pyrrole/dopamine (pyrrole 130 mM and dopamine 52 mM in PBS, pH 6) at a 0.5 V (vs. SCE) constant potential.
